# Sarcopenia as a risk factor of progression-free survival in patients with metastases: a systematic review and meta-analysis

**DOI:** 10.1186/s12885-023-10582-2

**Published:** 2023-02-07

**Authors:** Lingli Luo, Xiangru Shen, Shuai Fang, Teng Wan, Pan Liu, Peiling Li, Haifeng Tan, Yong Fu, Weiming Guo, Xiaojun Tang

**Affiliations:** 1 Medical College, Hunan Polytechnic of Environment and Biology, Hunan Province 421005 Hengyang, China; 2 Hengyang Medical College, University of South China, Hunan 421001 Hengyang, China; 3 Department of Neurology, Huazhong University of Science and Technology Union Shenzhen Hospital, Shenzhen, 518060 China; 4 Department of Trauma Orthopaedic, The Second Affiliated Hospital, Hengyang Medical College, University of South China, Hengyang, 421001 Hunan China; 5 Department of Sports Medicine, Huazhong University of Science and Technology Union Shenzhen Hospital, Shenzhen, 518060 China; 6 The Second Affiliated Hospital, Department of Spinal Surgery, Hengyang Medical School, University of South China, Hunan 421001 Hengyang, China

**Keywords:** Sarcopenia, Metastases, Progression-free survival, Meta, Retrospective study

## Abstract

**Background:**

Metastasis of cancer causes more than 90% of cancer deaths and is severely damaging to human health. In recent years, several studies have linked sarcopenia to shorter survival in patients with metastatic cancer. Several predictive models exist to predict mortality in patients with metastatic cancer, but have reported limited accuracy.

**Methods:**

We systematically searched Medline, EMBASE, and the Cochrane Library for articles published on or before October 14, 2022. Pooled Hazard Ratio (HR) estimates with 95% confidence intervals (CIs) were calculated using a random effects model. The primary outcome was an increased risk of death or tumor progression in patients with metastatic cancer, which is expressed as progression-free survival (PFS). In addition, we performed subgroup analyses and leave-one-out sensitivity analyses to explore the main sources of heterogeneity and the stability of the results.

**Results:**

Sixteen retrospective cohort studies with 1,675 patients were included in the 888 papers screened. The results showed that sarcopenia was associated with lower progression-free survival (HR = 1.56, 95% CI = 1.19–2.03, I2 = 76.3%, *P* < 0.001). This result was further confirmed by trim-and-fill procedures and leave-one-out sensitivity analysis.

**Conclusions:**

This study suggests that sarcopenia may be a risk factor for reduced progression-free survival in patients with metastatic cancer. Further studies are still needed to explain the reason for this high heterogeneity in outcome.

**Trial registration:**

CRD42022325910.

**Supplementary Information:**

The online version contains supplementary material available at 10.1186/s12885-023-10582-2.

## Background

Sarcopenia, from the Greek word "sarx" meaning "meat" and "penia" meaning loss, was first defined by Rosenberg in 1988 [1]. It is defined as a progressive, systemic skeletal muscle disease involving progressive and systemic loss of skeletal muscle mass and function [2, 3]. Sarcopenia is associated with a variety of diseases, including but not limited to natural aging, functional, metabolic and immune disorders, increased muscle catabolism due to cancer, and toxicity of anti-cancer treatments [2, 4, 5]. Previous studies have often confused cachexia with sarcopenia, a syndrome of weight loss and reduced muscle mass, and have been associated with poor prognosis [6, 7]. However, recent studies have shown that cachexia and sarcopenia represent different aspects of the muscle wasting spectrum [8, 9]. Approximately 15–50% of cancer patients with weight loss are sarcopenic rather than cachectic [10]. With regard to the prognosis of cancer patients, the U.S. Food and Drug Administration's criteria for the effectiveness of cancer drug trials are whether the survival of cancer patients is prolonged and whether clinical symptoms improve after treatment [11]. Although overall survival (OS) is the gold standard for evaluating clinical outcomes, the use of overall survival as a prognostic criterion for cancer patients may be biased. Possible reasons include the non-single nature of treatment for cancer patients from onset to end of life, the combination of other basic diseases and the use of other drugs during the course of the disease [12, 13]. Therefore, progression-free survival of tumor patients in clinical studies can be a better proxy for overall survival [14]. Progression-free survival is defined as the time from the time when a patient is randomly enrolled to the time when the patient is first proved to have tumor progression or death without tumor progression. It allows the trial data to be obtained over a relatively short follow-up period compared to OS as the trial endpoint. Progression-free survival to be the endpoint reduces the impact of subsequent treatments and is usually based on results obtained from objective and quantitative evaluations [15].

Many studies have been conducted to assess the predictive roles of sarcopenia in the occurrence of adverse events in cancer patients. Current systematic review suggests that sarcopenia negatively affects prognostic outcomes of cancer patients in terms of survival, physical activity, length of hospital stay and other complications [16–22]. However, we found that current studies were limited to specific primary tumors or site-specific metastatic cancers [23–25]. While sarcopenia as a systemic disease, we hypothesize that it is closely associated with the prognosis of multiple metastatic cancers. Metastasis causes greater than 90% of cancer death. Unlike primary tumors, which can often be cured using local surgery or radiation, metastasis is a systemic disease [26]. There is still controversy regarding the impact of sarcopenia on the prognosis and survival of patients with metastatic cancer. In the Lee et al. study, sarcopenia was not considered to be a potential factor in the patients' reduced PFS, which contradicts the findings of several other similar studies [27–29]. Therefore, to elucidate whether sarcopenia and progression-free survival have a potential relationship in patients with metastatic cancer, we performed a systematic review and meta-analysis of studies focusing on the relationship between sarcopenia and progression-free survival in patients with metastatic cancer.

## Methods

### Standard protocol approvals

This systematic review was conducted based on a predefined protocol and in accordance with Preferred Reporting Items for Systematic Reviews and Meta-Analyses (PRISMA) [30] and Meta-analysis of Observational Studies in Epidemiology (MOOSE) recommendations [31]. The review protocol was registered in PROSPERO with the registration unique identifying number (UIN) of CRD42022325910.

### Search strategy and selection criteria

Databases of Medline, Embase and Cochrane Central Register of Controlled Trials were systematically searched from inception to October 14, 2022 by two independent investigators (MZ and XS) without language or time restrictions. We used MeSH (for Medline and Cochrane)/Emtree (for Embase) terms combined with free-text words (including synonyms and closely related words) that were associated with metastases and sarcopenia.The detailed search strategy and specific terms were used, which were searched as free text words and as MeSH/Entrée terms without language restrictions. In addition, we also performed manual reference check of relevant articles, meta-analyses, reviews, and meeting abstracts. When two or more articles used the same cohort data, we preferred the most up-to-date ones with full-text information available. We perform study selection by a series of consecutive stages including duplicate checking using Endnote software, titles and abstracts screening, full-text article selection according to the eligibility criteria. These processes were conducted independently by two investigators (XS and TW). Conflicts were handled by consensus, and an adjudicator (WG) was consulted when necessary. If different opinions were encountered, senior experts would be consulted (SF or YF).

### Eligibility criteria

Studies were considered appropriate and were included in the analysis if they satisfied the following established inclusion criteria. [1] prospective or retrospective population-based cohort study design; [2] participants: Patients diagnosed with metastatic cancer, with or without sarcopenia. Sarcopenia was diagnosed according to the criteria given by EWGSOP in 2019 [2, 32]; [3] outcome: progression-free survival; [4] the measure of association: hazard ratio (HR) and corresponding 95% CIs provided from the original studies or having related data that could be used to calculate the risk ratios. We excluded hospital-based on community-based observational studies and those providing inadequate data to generate risk ratio for the association between sarcopenia and metastases.

### Study selection, data collection, and data extraction

Two investigators (XS and TW) independently read through, screened, extracted data from the included studies, and filled in the pre-designed data extraction excel forms. If there were any discrepancies, we would consult a third senior investigator (WG) until a consensus was reached. The following study characteristics were abstracted including study author, publication year, study design, study period, geographical region, observation period, population characteristics and age at cancer diagnosis, main treatment, measurements and definitions of sarcopenia, original cancer type, metastatic site of cancer and outcome.

### Quality assessment

The methodological quality for the included studies was evaluated using the Newcastle–Ottawa scale (NOS) [33] tool. It evaluates the cohort study through three modules and eight items in total, specifically, it includes the selection, comparability, and exposure/outcome evaluation of the study.. NOS uses the semi-quantitative principle of the star system to evaluate the quality of literature. Except for the maximum of 2 stars for comparability, the other items can be evaluated up to 1 star, with a full score of 9 stars. The higher the score, the higher the research quality.

### Statistical analysis

All statistical analyses were performed using Stata statistical software (version 15.1). The primary outcome was the PFS, defined as the length of time during and after the treatment of cancer, that a patient lives with the disease but the disease does not get worse. We applied the DerSimonian and Laird random effects meta-analysis to pool HRs along with the corresponding 95% CIs due to the anticipated substantial heterogeneity in terms of the enrolled populations [34]. To meta-analyze the HRs of PFS, we converted reported HRs to log HRs and used a generalized inverse variance method with a random effects model combining data. Results are reported with both effect estimates and 95% CIs. We used the I^2^ statistic to assess heterogeneity between studies, with I^2^ values > 50% indicating significant heterogeneity [35]. To explore the sources of heterogeneity, we carried out a series of subgroup analyses based on geographical regions (Europe and Asia), gender (male or female), original cancer type, variable analysis type of HR value (univariate vs multivariate), and methodological quality (low or high). Sensitivity analysis was performed by applying the leave-one-out method. Trim-and-fill technique is a simple funnel-plot-based method of testing and adjusting for publication bias in meta-analysis. funnel plot was used to detect publication bias in studies reporting overall survival, with a P-value < 0.1 indicating a significant difference [36].

## Results

### Literature search and study characteristics

**Fig. 1:**
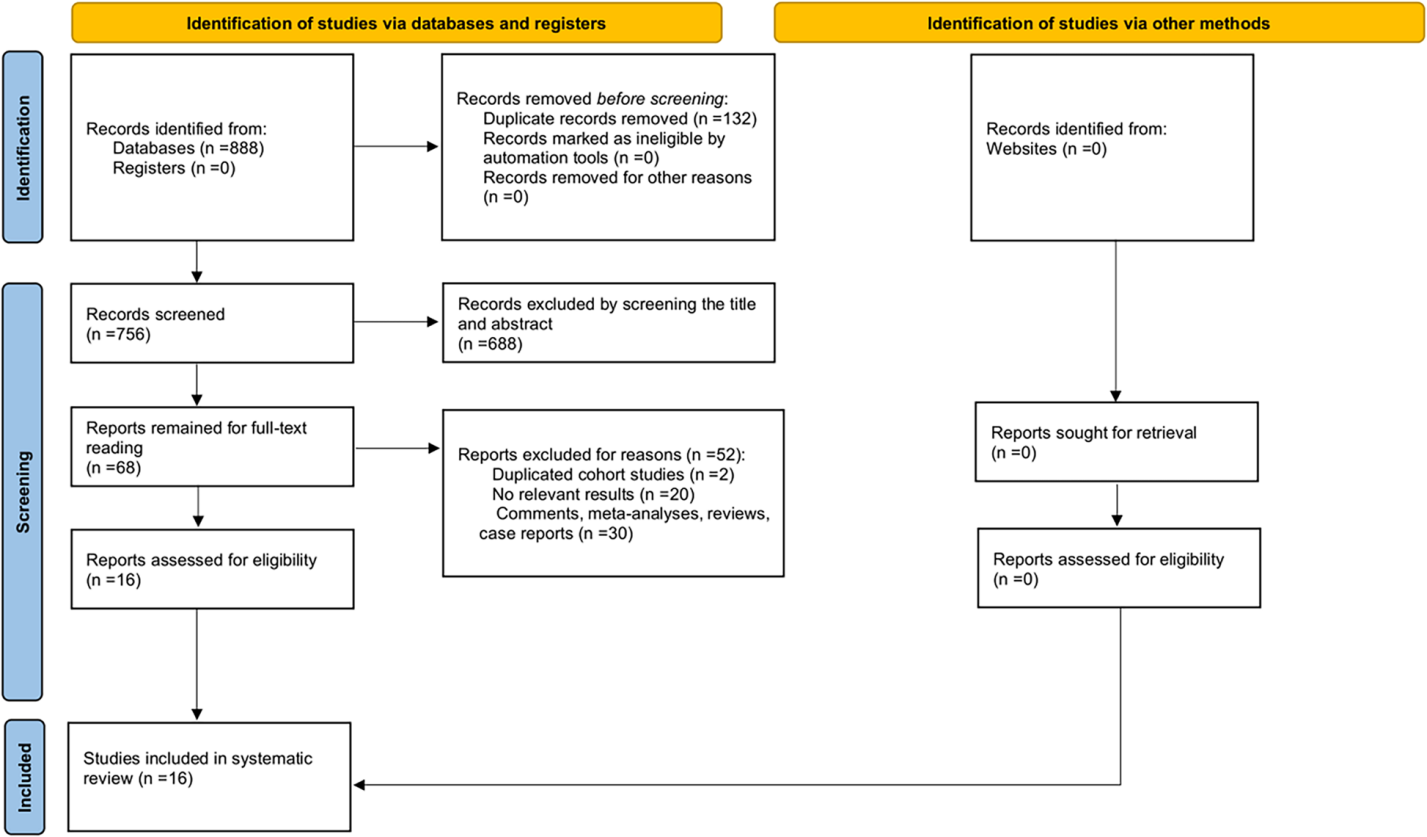
The flow chart of the literature selection

The initial literature search identified a total of 888 citations. After duplication removal, 756 studies remained for title and abstract review. During this process, we excluded 688 irrelevant citations and 68 potentially relevant studies were selected for full-text review. Due to non-population-based cohorts, reviews, meta-analyses or no outcome data reported, 16 studies [27–29, 37–49] involving 1,675 participants satisfied the inclusion criteria and were eligible to be included in the final meta-analysis (Fig. 1).

Table 1 presents the baseline characteristics of the included studies. Among the studies published between 20 16 and 2022, 8 studies [27–29, 37, 40, 42, 45, 46] were from Asia, 7 studies [39, 41, 43, 44, 47–49] were from Europe and a separate study [38] from Brazil. All of the studies were retrospective cohort, and 68.75% of the included studies (11/16) were of high quality with an NOS score of ≥ 7). The sample size of the included studies ranged from 29 to 353 participants. Original cancer type included hormone-sensitive prostate cancer, renal cell carcinoma, head cancer, lung cancer, renal cell cancer, bladder cancer, colorectal cancer, gastrointestinal stromal tumor, esophago–gastric junction cancer, proximal gastric cancer, distal gastric cancer, thyroid cancer, upper tract urothelial carcinoma, bile duct cancer, ampullary cancer, breast cancer and pancreatic cancer. 14 studies used the L3 skeletal muscle index (L3-SMI) to measure sarcopenia, 1 study used L3 psoas muscle index (L3-PMI) and 1 study used the total psoas area index (TPI). L3-SMI meant measuring the cross-section area of skeletal muscles (cm^2^) at L3 disc space divided by the square of the height of the patient (m^2^), the muscles are mainly the psoas major, and also include erector spinae, quadratus lumborum, transversus abdominis, external and internal oblique, and rectus abdominis muscles, for L3-PMI, the muscle is only the psoas; TPI meant measuring the total area of psoas area (cm^2^) divided by the square of the height of the patient (m^2^). For groups used L3-SMI, sarcopenia was defined as L3-SMI < 41 cm^2^/m^2^ in women, < 43 cm^2^/m^2^ in men with BMI < 25 kg/m^2^, and < 53 cm^2^/m^2^ in men with BMI > 25 kg/ m^2^; For group used L3-PMI, sarcopenia was defined as L3-PMI ≤ 6.36 cm^2^/m^2^ for men and ≤ 3.92 cm^2^/m^2^ for women; For group used TPI, sarcopenia was defined as TPI < 5.73 cm^2^/m^2^ for men and < 4.37 cm^2^/m^2^ for women.

**Table 1:** Characteristics of studies included in meta-analysis

First author	Year	Study design	Region	Diagnosed period	Sample size	Female%	Median/mean age	Main Treatment	Original cancer type	Cancer metastases
Lee, J. H	2021	Retrospective cohort study	Korea	2018 to 2021	70	0	66	Chemotherapy	Hormone-sensitive prostate cancer	Bone, lung, liver, pleura, adrenal gland, peritoneum, ureter
Lee, C. H	2021	Retrospective cohort study	Korea	2010 to 2017	78	24.4	61	Chemotherapy	Renal cell carcinoma	NR
Haik	2021	Retrospective cohort study	France	2013 to 2017	261	24	61.9 (mean)	Immunity therapy	Head, lung, renal, bladder cancer	Liver, lung, bone, brain
Gallois	2021	Retrospective cohort study	France	2013 to 2016	149	67	NR	Chemotherapy	Colorectal cancer	NR
Chang	2021	Retrospective cohort study	China	2007 to 2018	109	42.2	60.9 (mean)	Chemotherapy	Gastrointestinal stromal tumor	Liver
Catanese	2021	Retrospective cohort study	Italy	2010 to 2017	78	28.2	67	Chemotherapy	Esophago–gastric junction cancer, proximal gastric cancer, distal gastric cancer	Liver, lung, lymph nodes, peritoneum, bone
Yamazaki	2020	Retrospective cohort study	Japan	2015 to 2019	54	64.81	66.5	Chemotherapy	Thyroid cancer	Liver, lung, bone, brain, lymph node
Shimizu	2020	Retrospective cohort study	Japan	2017 to 2019	29	15	73	Chemotherapy	Bladder tumor, upper tract urothelial carcinoma	Liver, lung, lymph nodes, bone
Lee, B. M	2020	Retrospective cohort study	Korea	2007 to 2016	353	42.5	67	Chemotherapy	Gall bladder, intrahepatic bile duct, non-hilar bile duct, perihilar bile duct, ampullary cancer	NR
da Cunha	2019	Retrospective cohort study	Brazil	2009 to 2015	72	44.4	59.4 (mean)	Surgery and chemotherapy	Colorectal cancer	Peritoneum and other organs
Franzoi	2020	Retrospective cohort study	Belgium	2016 to 2019	50	100	61.2 (mean)	Chemotherapy	Breast cancer	Visceral disease, bone only or locorregional
Palleschi	2022	Retrospective cohort study	Italy	2009 to 2020	43	100	58	Chemotherapy	Breast cancer	NR
Williet	2021	Retrospective cohort study	France	2012 to 2018	79	45.6	66	Chemotherapy	Pancreatic cancer	NR
Ishihara	2016	Retrospective cohort study	Japan	2007 to 2014	71	29.6	64	Chemotherapy	Renal Cell Carcinoma	NR
Gu	2017	Retrospective cohort study	China	2008 to 2014	101	35.6	59	Chemotherapy	Renal cell carcinoma	NR
Malik	2021	Retrospective cohort study	Poland	2017 to 2020	78	45	64.5	Chemotherapy	Colorectal cancer	Liver, lung, lymph node, peritoneum

**First author**	**Measurements of sarcopenia**	**Sarcopenia definition**	**Outcomes (HR, 95% CI)**	**median/mean Follow-up period **
Lee, J. H.	L3-SMI	SMI ≤ 52.4 cm2/m2	PFS(crude HR, 4.73, 1.40–15.96; adjusted HR, 3.77, 0.95–14.99)	20.5 months
Lee, C. H.	L3-SMI	SMI of < 43cm2/m2 and < 53 cm2/m2 for men with a BMI of < 25 kg/m2 and ≥ 25kg/m2, respectively, and < 41 cm2/m2 for women	PFS(crude HR, 3.18, 1.84–5.47; adjusted HR, 2.62, 1.47–4.66)	15.4 months
Haik	L3-SMI	< 41 cm2/m2 for females and < 43 cm2/m2 for males if body mass index (BMI) < 25 kg/m2 or < 53 cm2/m2 if BMI ≥ 25 kg/m2	PFS(adjusted HR, 0.80, 0.60–1.055)	NR
Gallois	L3-SMI	men < 40.3 cm2/m2 and women < 32.0 cm2/m2	PFS(crude HR, 1.5, 1.0−2.2)	23 months
Chang	L3-PMI	< 6.36 cm2/m2 for males and < 3.92 cm2/m2 for females	PFS(adjusted HR, 2.333, 1.251−4.349)	NR
Catanese	L3-SMI	male patients asSMI < 43 cm2/m2 if BMI < 25 kg/m2 and SMI < 53 cm2/m2 if BMI ≥ 25 kg/m2, and infemale patients as SMI < 41 cm2/m2 irrespective of BMI	PFS(crude HR, 0.83, 0.53–1.32)	52.2 months
Yamazaki	L3-SMI	< 42 cm2/m2 for males and < 38 cm2/m2 for females	PFS(adjusted HR, 2.488, 1.058–5.846)	NR
Shimizu	L3-PMI	≤ 6.36 cm2/m2 for men and ≤ 3.92 cm2/m2 for women	PFS(crude HR, 2.99, 1.14–7.85; adjusted HR, 2.79, 1.14–7.32)	7 months
Lee, B. M.	L3-SMI	< 55 cm2/m2 for male and < 39 cm2/m2 for female	PFS(crude HR, 0.75, 0.61–0.93)	7.77 months
da Cunha	L3-SMI	SMI < 41 cm2/m2 for women; SMI < 43 cm2/m2 if BMI < 25 kg/m2 and SMI < 53 cm2/m2 if BMI ≤ 25 kg/m2 for men	PFS(crude HR, 2.34, 1.40–3.93; adjusted HR, 1.78, 1.00–3.14)	23.6 months
Franzoi	L3-SMI	SMI < 40 cm2/m2	PFS(crude HR, 2.52, 1.02–6.19)	14.4 months
Palleschi	L3-SMI	SMI < 40 cm2/m2	PFS(crude HR, 0.98, 0.47–2.03)	33 months
Williet	TPI	< 5.73 cm2/m2 for men and < 4.37 cm2/m2 for women	PFS(crude HR, 2.3, 1.38–3.85; adjusted HR, 2.04, 1.18–3.53)	NR
Ishihara	L3-SMI	men with a BMI of < 25 kg/m2 and SMI < 43 cm2/m2; men with a BMI of > 25 kg/m2 and SMI < 53 cm2/m2; women with SMI < 41 cm2/m2	PFS(crude HR, 3.15, 1.66–6.41; adjusted HR, 2.54, 1.19–5.65)	20.2 months (mean)
Gu	L3-SMI	< 40.8 cm2/m2 for males and < 34.9 cm2/m2 for females	PFS(crude HR, 1.426, 0.880–2.310)	NR
Malik	L3-SMI	< 52.4 cm2/m2 for men and < 38.5 cm2/m2 for women	PFS(adjusted HR, 1.47, 0.88–2.5)	19.1 months

*L3* 3rd lumbar spine, *SMI* skeletal muscle index, *PMI* psoas muscle index, *BMI* body Mass Index, *TPI* The total psoas area index, *PFS* progression-free survival. *HR* Hazard Ratio

### Methodological quality (risk of bias)

Using the NOS tool for cohort studies, a total of 5 studies [37, 40, 42, 44, 48] had a high risk of bias, with each study having 2 to 3 possible sources of bias, bias was most common in adequacy of follow-up. A total of 9 scores were assigned to 9 item questions, and a score of less than 7 was defined as high risk of bias (Table 2).

**Table 2:** Methodological quality score of the included studies based on the Newcastle–Ottawa scale (NOS) tool

Author	Year	Study Design	Selection				Comparability	Exposure/Outcome			Total Score	Risk of Bias
			Representativeness of cohort *	Selection of control cohort *	Ascertainment of exposure *	Outcome not present at start *	Comparability of cohorts **	Assessment of outcome *	Length of follow-up *	Adequacy of follow-up *	**Total score**	
Lee, J. H	2021	Retrospective cohort study		*	*	*	**	*	*	*	8	Low
Lee, C. H	2021	Retrospective cohort study		*	*	*	**	*	*	*	8	Low
Haik	2021	Retrospective cohort study		*	*	*	**	*			6	High
Gallois	2021	Retrospective cohort study		*	*	*	**	*	*	*	8	Low
Chang	2021	Retrospective cohort study		*	*	*	**	*			6	High
Catanese	2021	Retrospective cohort study		*	*	*	**	*	*		7	Low
Yamazaki	2020	Retrospective cohort study		*	*	*	**	*			6	High
Shimizu	2020	Retrospective cohort study		*	*	*	**	*	*	*	8	Low
Lee, B. M	2020	Retrospective cohort study		*	*	*	**	*	*	*	8	Low
da Cunha	2019	Retrospective cohort study		*	*	*	**	*	*	*	8	Low
Franzoi	2020	Retrospective cohort study		*	*	*	**	*	*	*	8	Low
Palleschi	2022	Retrospective cohort study		*	*	*	**	*	*	*	8	Low
Williet	2021	Retrospective cohort study		*	*	*	**	*			6	High
Ishihara	2016	Retrospective cohort study		*	*	*	**	*	*	*	8	Low
Gu	2017	Retrospective cohort study		*	*	*	**	*			6	High
Malik	2021	Retrospective cohort study		*	*	*	**	*	*	*	8	Low

### Associations between sarcopenia and the risk of poor PFS

**Fig. 2:**
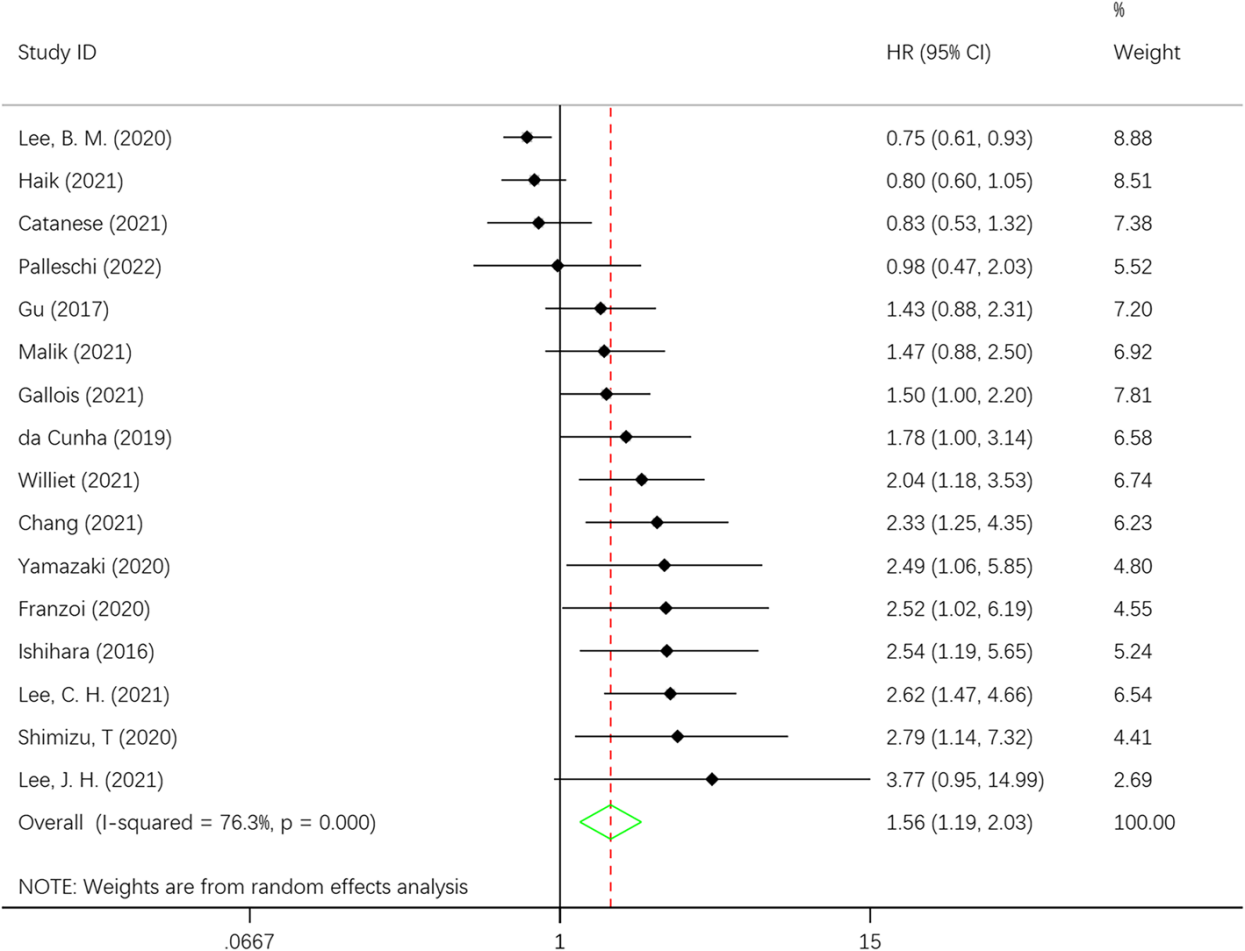
Hazard risk (HR) for association of sarcopenia with decrease of progression-free survival in patients with metastases

When we meta-analyzed the 16 studies, the results showed that the pooled HR of progression-free survival reached 1.56 (95% CI = 1.19–2.03) in all site cancer survivors compared with noncancer controls. Heterogeneity among studies was high (I2 = 76.3%; *P* < 0.001) (Fig. 2).

#### Subgroup analysis

In the subgroup analysis, we found that the risk of reduced PFS was significant in two subgroups: the Asian population (HR 1.98, 1.18–3.31, I^2^ = 83.6%) and the study with low risk of bias (HR 1.56, 1.10–2.21, I^2^ = 77.1%). In addition, subgroup analysis did not show a significant correlation between patient gender and lower PFS (Table 3). For specific cancer sites, we noted a significant correlation between lower PFS and urologic cancer (HR 1.57, 1.02–2.41, I^2^ = 7.3%), endocrine gland cancer (HR2.16, 1.36–3.43, I^2^ = 0). After observing and categorizing the gastrointestinal cancer subgroup, we found that colorectal cancer (HR 1.55, 1.18–2.04, I^2^ = 79.9%) was significantly associated with lower PFS, while gastric cancer did not show an increased risk of lower PFS. Interestingly, in the analysis of variable types, we found that the results obtained from univariate variables were contrary to the unified results (HR 0.84, 0.70 to 0.98, I^2^ = 50.9). In addition, the heterogeneity of the results obtained from multivariate variables also decreased (HR 1.01, 0.81 to 1.22, I^2^ = 57.8), This suggests that the data type of the original article may be the cause of the high heterogeneity (Table 3).

**Table 3:** Subgroup analyses for the effect of sarcopenia on PFS in patients with metastases

**Variables**		**HR**	**95% CI**	**I**^**2**^ **(%)**	**No. studies**	***P*** ** for interaction**
**Regions**						< 0.001
	Asia	1.98	1.18 to 3.31	83.6	8	
	Europe	1.25	0.91 to 1.73	66.9	7	
**Gender**						< 0.001
	Female ≤ 50%	1.53	1.12 to 2.11	79.8	12	
	Female > 50%	1.61	1.11 to 2.33	21.4	4	
**Original cancer type**						< 0.001
	Gastrointestinal	1.28	0.87 to 1.89	79.9	6	
	Urologic	1.57	1.02 to 2.41	7.3	5	
	Endocrine Gland	2.16	1.36 to 3.43	0	2	
	Breast	1.51	0.60 to 3.80	0	2	
**Quality assessment**						< 0.001
	Low risk of bias	1.56	1.10 to 2.21	77.1	11	
	High risk of bias	1.59	0.96 to 2.62	79.2	5	
Type of HR						< 0.001
	Univariate	0.84	0.70 to 0.98	50.9	6	
	Multivariate	1.01	0.81 to 1.22	57.8	10	

#### Sensitivity analyses and publication bias

**Fig. 3:**
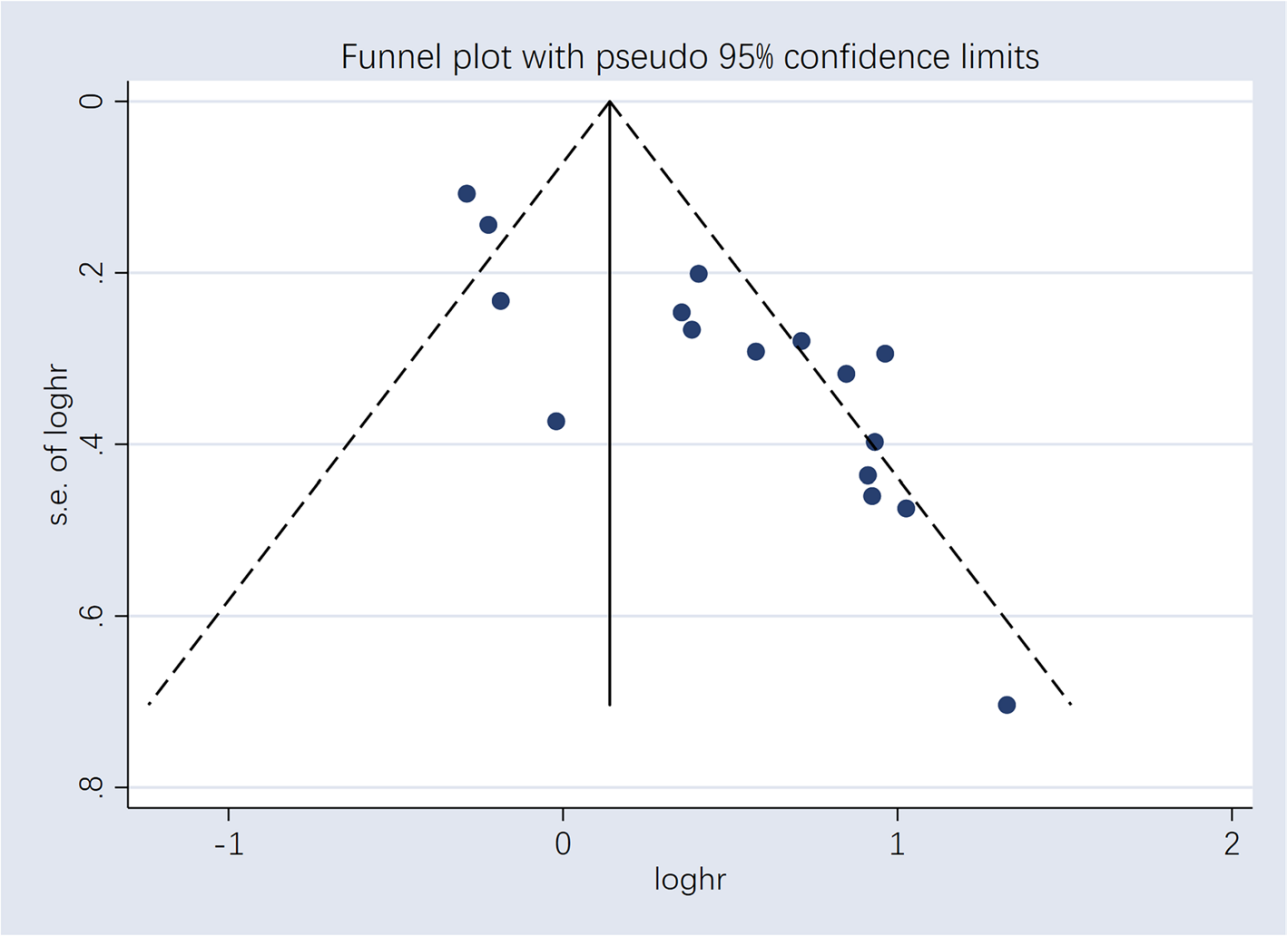
Funnel plots assessing the potential impact of publication bias

Sensitivity analyses were performed using the leave-one-out method to further examine the stability of the result. We found some studies that significantly changed the pooled HR (lowest HR 1.19, 0.92–1.47; highest HR 1.41, 1.07–1.76), and after a careful reading of the included articles and excluding one low-quality study, we obtained robust results (lowest HR 1.36, 1.01–1.70; highest HR 1.58, 1.15–2.00) [44]. Visual inspection of the funnel plot for the outcome revealed asymmetry, indicating potential evidence of publication bias. Both Begg’s test (*p* = 0.027) and Egger’s test (*p* < 0.001) were significant, therefore publication bias is likely to be the underlying cause of asymmetry. Trim-and-fill technique adjusted for publication bias, found funnel plot region contained only two potentially missing studies, but all were located at the bottom of the funnel plot [50]. After adjusting for publication bias, HR = 1.472, 95% CI 1.140–1.901, which is consistent with our previous results, indicating that our results are still reliable (Fig. 3) (Table 4).

**Table 4:** Subgroup analyses for Gastrointestinal Neoplasms

Cancer type	HR	95% CI	I^2^ (%)	No. studies
Colorectal	1.55	1.18 to 2.04	0	3
Gastric	1.36	0.49 to 3.74	85.5	2

## Discussion

### Principal findings

This systematic review and meta-analysis of 16 population-based cohort studies demonstrated a statistically significant increased risk of reduced PFS in metastatic cancer patients with concomitant sarcopenia compared with non-sarcopenic patients, with results remaining consistent after adjusting for potential publication bias. Furthermore, our results suggest that sarcopenia has a stronger contribution to worsening PFS in Asian populations, a finding that is stable in high-quality studies. Our findings regarding the association between sarcopenia and the risk of worsening PFS in patients with metastatic cancer are consistent with a systematic review and multiple clinical studies, all of which suggest that patients with sarcopenia have a risk of worsening PFS [48, 51, 52]. However, the results of most of these studies were from cohorts with high limitations. These studies were limited to specific cancer types and cancer patients treated with chemotherapy or radiation (e.g., patients with metastatic renal cell carcinoma treated with cabozantinib). To our knowledge, this study is one of the few studies involving a representative population of multiple cancer types, with meta-analysis and systematic review from high-quality population-based cohort studies, rather than previous individual or narrative studies.

### Potential mechanisms

Originally used to describe the loss of muscle mass with age, the European Working Group on Sarcopenia in Older People (EWGSOP) has recently defined sarcopenia to include impaired muscle strength and poor physical performance [2]. Previously, most people considered sarcopenia as an inevitable part of aging. However, the degree of sarcopenia is highly variable and depends on the presence of certain risk factors, such as lack of exercise, age-related decreases in hormone concentrations and cytokine imbalances, decreased ability to synthesize proteins, failure of satellite cell activation, potential effects of microRNA, oxidative stress due to chronic diseases, etc. [53–57]. There are two broad types of sarcopenias, primary sarcopenia caused by aging and secondary sarcopenia mainly caused by malignancy [58]. In cancer patients, the adverse effects of sarcopenia include increased susceptibility to adverse events, increased complications from cancer surgery and chemotherapy toxicity, and difficulty in pursuing further cancer-directed therapy [59]. Sarcopenia is characterized by a decrease in both muscle strength and mass, and therefore may increase the risk of falls and fractures in older adults [60, 61]. In addition, decreased muscle function can affect swallowing and breathing, which can aggravate or cause respiratory disease [62]. At the same time, we note that sarcopenia may interact with certain underlying diseases in patients, leading to a poor prognosis. In one study, it was confirmed that patients with COPD are often associated with sarcopenia and negatively affect important clinical outcomes [63]. For the heart, sarcopenia is thought to be closely associated with heart failure (HF) [64]。Sarcopenia may promote HF development through different mechanisms, including pathological ergoreflex [64, 65]. At the same time, HF may induce sarcopenia through multiple pathways, such as hormonal changes, poor nutrition, lack of exercise and etc. Therefore, early identification and intervention for the basic diseases in patients with metastatic cancer may be necessary in the future.

It is now generally accepted that chemotherapy is also a cause of sarcopenia in cancer patients [66–68]. However, the relationship between the two is equally complex. One study found that patients with metastatic colorectal cancer (mCRC) had a significant reduction in muscle area during chemotherapy, and the rate of muscle loss observed in the study was 24 times faster than the normal rate of muscle loss (1% per year) [69]. Another study of adjuvant chemotherapy in patients with colon cancer found that baseline sarcopenia was associated with an increased incidence of all grade 3–4 chemotherapy-induced toxicities [70]. In addition, in patients with colon, lung, esophageal, gastric, and other types of cancer, mortality increases and progression-free survival decreases after chemotherapy, even if patients with sarcopenia do not exhibit lower overall survival [70–73]. The current explanation for this phenomenon is that patients with sarcopenia are forced to reduce doses or delay dosing cycles due to excessive toxicity to oncology treatment [74]. It is common practice to base chemotherapy on the body surface area of each patient, without taking into account the large and unpredictable fraction of body weight accounted for by adipose tissue [75]. A large amount of evidence shows that this method fails especially in patients with sarcopenia, and the related toxicity risks cannot be solved [76–78]. Obviously, although some physiological constants may be related to body surface area, they are not related to other anthropometric parameters, such as body mass index. Besides, sarcopenia also reduces the effective effect of chemotherapy. Studies have shown that among breast cancer patients with sarcopenia, many chemotherapeutic drugs such as capecitabine, paclitaxel, docetaxel, and nab-paclitaxel have poor effects [74, 79]. The reasonable explanation may be that the adverse outcome may be related to the high toxicity rate, which in turn may lead to the necessary dose reduction and the provision of effective tumor treatment at a lower dose, thus reducing the therapeutic benefit [80]. To sum up, cancer patients are prone to sarcopenia before and after chemotherapy, and sarcopenia occurring or aggravated during chemotherapy will worsen the prognosis and aggravate the toxicity caused by chemotherapy. Therefore, chemotherapy cycles and doses need to be carefully set based on drug toxicity and therapeutic effects.

In our study, we found that the risk of sarcopenia and reduced PFS was not significant in the European population. It was found that, with the exception of the study of Haik, the remaining studies that showed unstable results were low risk of bias studies, and sensitivity analysis also yielded relatively stable results after excluding this study [44]. All patients in this study were from a single hospital cohort and did not target a specific cancer, but rather included multiple cancers. In addition, this study was the only cohort to use immune checkpoint therapy on patients, which has relatively few side effects. Other studies have commonly used chemotherapy, and patients may have been treated with the previously described, mutually reinforcing effect of sarcopenia and chemotherapy toxicity, but this was not a problem in immunotherapy. This could be a potential reason why this research concluded that sarcopenia was not significantly associated with PFS. Several studies have now demonstrated that cancer patients treated with immune checkpoint inhibitors alone or in combination with chemotherapy have significantly improved survival compared to chemotherapy alone [81–83]. Whether immunotherapy exerts a better improvement in PFS in patients with sarcopenia needs to be further explored in more clinical studies.

### Implications

This meta-analysis provided important future clinical implications for the risk of worsening PFS in metastatic cancer patients with concomitant sarcopenia, yielding an approximate risk estimate with an HR of 1.56 (95% CI = 1.19–2.03) for PFS. Early screening and effective interventions are clinically important in the prevention and treatment of sarcopenia. Second, optimal strategies for prevention and management of sarcopenia have not been established due to the widespread neglect of sarcopenia in cancer. Due to the lack of reliable clinical data to guide clinicians, physicians may consider the use of specific treatment options based on the history of adjuvant therapy (e.g., chemotherapy or radiation therapy), baseline information, etc., in cancer patients. In addition, screening and treatment of individuals at high risk for stroke has implications for the prevention of sarcopenia and for reducing the burden of sarcopenia in the general elderly population.

### Strengths and limitations

The current study has several advantages in the following aspects. First, the current systematic evaluation and meta-analysis includes a more representative population in the relevant field, providing up-to-date evidence on the association between the risk of worsening PFS in metastatic cancer patients with concomitant sarcopenia. Second, we developed a systematic and comprehensive database search strategy based on the major online databases (Medline, Embase, and Cochrane Library) with no search date restrictions so that we could retrieve as many relevant articles from around the world as possible, avoiding publication bias on pooled results and improving the reproducibility of results. Third, almost all included studies were from national cohorts or population-based cohorts, thus minimizing potential selection bias stemming from study design. In addition, a transparent methodological quality assessment of the included studies was performed using the NOS list for cohort studies. Fourth, several methods, including subgroup analysis and sensitivity analysis, have been applied to thoroughly identify sources of heterogeneity based on abstract study-level baseline characteristics. In these sensitivity analyses, after excluding one low-quality study, our results remained stable and Egger's test combined with the cut-and-patch method found no evidence of publication bias.

This study also has some limitations. First, significant heterogeneity was found in the included studies, which was predictable and may be partly due to differences in baseline characteristics of the population (sex, race, tumor primary site and metastatic site, etc.), exposure to treatment (adjuvant chemotherapy, radiation therapy, or hormonal therapy), and statistical methods (adjustment for confounders). Although several methods were applied to adjust for outcomes, considerable moderate to high heterogeneity remained. After careful study, we concluded that irregularities in PFS can introduce bias in the results, mainly due to differences in the interval between patient follow-up after treatment. For PFS, PFS is susceptible to the influence of the follow-up interval because the exact time point at which a patient progresses is uncertain. Different follow-up intervals may lead to highly variable trial results. For example, if patients are asked to follow up every 3 months, assuming a high degree of patient compliance, and if patients in the trial group have a prolonged survival of less than 3 months compared to the control group, it cannot be concluded that the trial group is better than the control group because tumor progression in both groups will be detected at the same review every 3 months, then PFS is recorded as No difference. In a more extreme case, if the tumor is reviewed every 3 months after treatment, and the tumor is determined to be progression-free at the first post-treatment review (3 months) in the trial group, but progression appears soon after the review (much less than 3 months, or even days) but is not detected, while tumor progression is detected at the first post-treatment review (nearly 3 months) in the control group, the difference in progression-free survival between the two groups is considered to be 3 months, while the true difference between the two groups is close to 0. The true difference is close to 0, which would result in a 3-month bias. Such differences can occur not only within studies, but also between studies due to inconsistent PFS intervals, and the shorter the follow-up interval for patients, the more reliable their trial results. This inference is supported by the fact that most of our studies did not mention the follow-up interval and that those that reported this element were at low risk of bias and had a higher degree of confidence, as other investigators have also mentioned this directly or indirectly [84]. However, given that the results of most subgroup analyses and sensitivity analyses were highly consistent with the primary outcome, we believe that the impact of these heterogeneities on the primary outcome of the study is limited. Second, the results of this meta-analysis are based on observational cohort studies, which may be limited by confounding factors such as patient gender, adjuvant treatment modality, and follow-up interval. However, all included studies provided data comparing sarcopenic and non-sarcopenic populations and matched with a number of important covariates suggesting a consistently increased risk of worsening PFS in sarcopenic patients. Third, because our study was a single-study-level meta-analysis rather than an individual patient-level meta-analysis, we were unable to perform more detailed subgroup analyses (e.g., risk analyses based on events during cancer survival and follow-up) and were unable to explore progressive sarcopenia that occurred during follow-up. Fourth, some subgroup analyses found nonsignificant results, which we believe may be due to the relatively small sample size and low statistical efficacy. More evidence from high-quality prospective cohort studies on the impact of sarcopenia on the risk of death or progression in patients with metastatic cancer is needed. Finally, the meta-analysis was limited to studies published in peer-reviewed journals in English. We may have missed articles published in other languages or in journals outside of the three databases we searched. In addition, unpublished gray literature was not included. However, three major databases, Medline, Embase, and the Cochrane Library, published the vast majority of available reports. Despite these limitations, the current study includes the vast majority of cancer types, which provides a largely adequate sample size for meaningful and robust statistical analyses.

## Conclusions

In this article, we performed a systematic evaluation and meta-analysis of sarcopenia in metastatic cancer patients. The results suggest that sarcopenia might be an indicator of reduced progression-free survival in metastatic cancer patients. However, there is still a need to conduct larger prospective cohort studies to confirm the conclusion.

## Supplementary Information

**Additional file 1: Text S1.** Search strategy Database: Pubmed from inception to Present> (Search date: October 14, 2022). **Text S2.** Search strategy. Database:EMBASE (Search date: October 14, 2022). **Text S3.** Search strategy. Database: Cochrane Library from inception to Present> (Search date: October 14, 2022).

## Data Availability

All data generated or analysed during this study are included in this published article.

## Abbreviations

HR: Hazard Ratio
CIs: Confidence intervals
OS: Overall survival
PFS: Progression-free survival
NOS: Newcastle–Ottawa scale
L3-SMI: L3 skeletal muscle index
L3-PMI: L3 psoas muscle index
TPI: Total psoas area index
mCRC: Metastatic colorectal cancer
