# Supplementary material for: Sarcopenia as a risk factor of progression-free survival in patients with metastases: a systematic review and meta-analysis
Source: BMC Cancer. 2023 Feb 7;23:127. doi: 10.1186/s12885-023-10582-2 (PMC9906917; doi:10.1186/s12885-023-10582-2)
Supplement: Supplementary file 1 — Additional file 1: Text S1. Search strategy Database: Pubmed from inception to Present> (Search date: October 14, 2022). Text S2. Search strategy. Database:EMBASE (Search date: October 14, 2022). Text S3. Search strategy. Database: Cochrane Library from inception to Present> (Search date: October 14, 2022). [file 12885_2023_10582_MOESM1_ESM.docx]

**Table 3**

**Text S1 Search strategy**

**Database: Pubmed from inception to Present> (Search date: October 14, 2022)**

--------------------------------------------------------------------------------

***Sarcopenia terms:***

1. "Sarcopenia"[Mesh]
2. "Muscle Strength"[Mesh]
3. "Physical Fitness"[Mesh]
4. "Geriatric Assessment"[Mesh]
5. (Morphometrics or Physical performance or Frail* or Geriatric Assessment or Physical function or Muscle strength or Muscle function or Hand grip strength or Gait speed or Walking speed or Fitness or Physical fitness or Body composition or Sarcopeni* or Cachexia or Skeletal muscle or Muscle mass) [Title/Abstract]
6. 1-5/or

***metastases terms:***

1. "Neoplasm Metastasis"[Mesh]
2. (Metastatic or Neoplasm Metastases or Metastas*) [Title/Abstract]
3. 7-8/or

***Progression-free survival terms:***

1. "Progression-Free Survival"[Mesh]
2. (Progression Free Survival or Survival, Progression-Free or Event Free Survival or Survival, Event-Free) [Title/Abstract]
3. 10-11/or

***Final search results: Combining Sarcopenia and Spinal metastases:***

1. 6 and 9 and 12 (177)

**Text S2 Search strategy**

**Database: EMBASE (Search date: October 14, 2022)**

--------------------------------------------------------------------------------

***Sarcopenia terms:***

1. 'sarcopenia'/exp
2. 'muscle strength'/exp-
3. 'fitness'/exp
4. 'geriatric assessment'/exp
5. (morphometrics:ab,ti OR 'physical performance':ab,ti OR frail*:ab,ti OR 'geriatric assessment':ab,ti OR 'physical function':ab,ti OR 'muscle strength':ab,ti OR 'muscle function':ab,ti OR 'hand grip strength':ab,ti OR 'gait speed':ab,ti OR 'walking speed':ab,ti OR fitness:ab,ti OR 'physical fitness':ab,ti OR 'body composition':ab,ti OR sarcopeni*:ab,ti OR cachexia:ab,ti OR 'muscle mass':ab,ti OR 'skeletal muscle':ab,ti) AND [embase]/lim
6. 1-5/or

***metastases terms:***

1. 'metastasis'/exp
2. (metastatic:ab,ti OR 'neoplasm metastases':ab,ti OR metastas*:ab,ti) AND [embase]/lim
3. 7-8/or

***Progression-free survival terms:***

1. 'progression free survival'/exp
2. ('progression free survival'/exp OR 'progression free survival' OR 'survival, progression-free' OR 'event free survival'/exp OR 'event free survival' OR 'survival, event-free':ti,ab) AND [embase]/lim
3. #10 OR #11

***Final search results: Combining Sarcopenia and Spinal metastases:***

1. 6 and 9 and 12 (693)

**Text S3 Search strategy**

**Database: Cochrane Library from inception to Present> (Search date: October 14, 2022)**

--------------------------------------------------------------------------------

***Sarcopenia terms:***

#1 MeSH descriptor: [Muscle Strength] explode all trees

#2 MeSH descriptor: [Physical Fitness] explode all trees

#3 MeSH descriptor: [Geriatric Assessment] explode all trees

#4 MeSH descriptor: [Sarcopenia] explode all trees

#5 (Morphometrics or Physical performance or Frail* or Geriatric Assessment or Physical function or Muscle strength or Muscle function or Hand grip strength or Gait speed or Walking speed or Fitness or Physical fitness or Body composition or Sarcopeni* or Cachexia or Skeletal muscle or Muscle mass or Skeletal muscle):ti,ab,kw (Word variations have been searched)

#6 #1 or #2 or #3 or #4 or #5

***metastases terms:***

#7 MeSH descriptor: [Neoplasm Metastasis] explode all trees

#8 (Metastatic or Neoplasm Metastases or Metastas*):ti,ab,kw (Word variations have been searched)

#9 #7 or #8

***Progression-free survival terms:***

#10 MeSH descriptor: [Progression-Free Survival] explode all trees

#11 (Progression Free Survival or Survival, Progression-Free or Event Free Survival or Survival, Event-Free):ti,ab,kw (Word variations have been searched)

#12 #10 #11

***Final search results: Combining Sarcopenia and Spinal metastases:***

#10 #6 and #9 and #12 (18)
